# Extended-spectrum β-lactamase *bla*_CTX-M-1_ group in gram-negative bacteria colonizing patients admitted at Mazimbu hospital and Morogoro Regional hospital in Morogoro, Tanzania

**DOI:** 10.1186/s13104-021-05495-x

**Published:** 2021-02-27

**Authors:** Nyambura Moremi, Vitus Silago, Erick G. Mselewa, Ashery P. Chifwaguzi, Mariam M. Mirambo, Martha F. Mushi, Lucas Matemba, Jeremiah Seni, Stephen E. Mshana

**Affiliations:** 1 Quality Assurance & Training Centre, National Health Laboratory, P. O. Box 9083, Dar Es Salaam, Tanzania; 2 Department of Microbiology and Immunology, Weill Bugando School of Medicine, Catholic University of Health and Allied Sciences-Bugando, P. O. Box 1464, Mwanza, Tanzania; 3 National Institute for Medical Research, P. O. Box 805, Dodoma, Tanzania

**Keywords:** Antimicrobial stewardship, ESBL colonization, ESBL genes, Infection prevention and control

## Abstract

**Objective:**

The objective of this study was to determine the proportion of extended spectrum β-lactamase producing gram-negative bacteria (ESBL-GNB) colonizing patients admitted at Mazimbu hospital and Morogoro Regional hospital, in Morogoro, Tanzania. Rectal colonization with ESBL-GNB increases the risks of developing bacterial infections by extra-intestinal pathogenic ESBL-GNB.

**Results:**

Of the 285 patients investigated, 123 (43.2%) carried ESBL-GNB in their intestines. Five of the 123 ESBL positive patients were colonized with two different bacteria, making a total of 128 ESBL producing isolates. *Escherichia coli* (n = 95, 74.2%) formed the majority of ESBL isolates. The proportion of CTX-M-1 group genes among ESBL isolates tested was 94.9% (93/98). History of antibiotic use (OR: 1.83, 95% CI: 1.1–3.2, P = 0.03), being on antibiotic treatment (OR: 2.61, 95% CI: 1.5–4.53, P = 0.001), duration of hospital stay (OR: 1.2, 95% CI: 1.1–1.3, P < 0.001) and history of previous admission (OR: 2.24, 95% CI: 1.2–4.1, P = 0.009) independently predicted ESBL-GNB carriage.

## Introduction

Extended spectrum beta-lactamases (ESBLs) production, is the commonest mechanism of resistance to multiple broad-spectrum beta-lactams among gram-negative bacteria mainly members of the family Enterobacteriaceae [1, 2]. ESBL enzymes hydrolyze beta-lactam ring of the beta-lactams making these antibiotics ineffective against ESBL producing bacteria [3]. The *bla*_CTX-M_ group out of other ESBL groups, is the commonest reported group of ESBL genes in different part of the World including in Tanzania [2, 4–7]. CTX-M enzymes effectively hydrolyzes third generation cephalosporins (3GCs) e.g., ceftriaxone and cefotaxime but not oxyimino-cephalosporins e.g., ceftazidime [8]. Although, some CTX-M members; CTX-M-15, -16 and -19 have been reported to hydrolyze ceftazidime activity [9–11].

Colonization with ESBL producing gram-negative bacteria (ESBL-GNB) increases the risk of developing multidrug resistant (MDR) bacterial infections e.g., bloodstream infection, urinary tract infection or wound infection [12]. Infections with MDR bacteria are associated with increased days of hospitalization, healthcare costs and mortalities from treatment failure and/or limited therapeutic options [13].

In Tanzania, previous studies from national and zonal referral hospitals have reported magnitudes of rectal/intestinal carriage of ESBL producing gram-negative bacteria (ESBL-GNB) ranging from 15% to 59.7% among hospitalized patients [14–17]. ESBL producing *E. coli* (ESBL-EC) and ESBL producing *K. pneumoniae* (ESBL-KP) are frequently reported with proportion ranging from 30% to 68.7% and 28.2% to 77.1%, respectively [14, 15, 17]. The magnitude of ESBL rectal colonization and associated factors among hospitalized patients in other tiers of the healthcare facilities like regional and district hospitals has not been well studied in developing countries including Tanzania. The objectives of this study was to determine the magnitude and factors associated with rectal colonization with ESBL producing gram-negative bacteria (ESBL-GNB) among hospitalized patients at Mazimbu hospital and Morogoro Regional hospital in Morogoro, Tanzania. Therefore, this study’s findings provide baseline information to improve measures of infections prevention and control (IPC).

## Main text

### Methods

#### Study design, population, duration and settings

This cross-sectional analytical study was conducted among patients admitted at Mazimbu hospital (~ 30 beds capacity) and Morogoro Regional hospital (~ 450 beds capacity) in Morogoro region, Tanzania between May and July 2017. A minimum sample size of 280 was obtained using Kish and Leslie formula (1965) and a prevalence of 24% [5]. Participants stayed ≥ 24 h in hospital wards were eligible to be enrolled in this study. A standardized data collection tool was used to collect socio-demographic and clinical associated data relevant to study’s objectives.

#### Sample collections and laboratory procedures

Sterile swabs (Mast Diagnostica GmbH, Germany) in Amies transport media were used to collect a single time rectal swab from participants. Then, transported to Microbiology laboratory at Morogoro Regional hospital within 4 h of collection for laboratory analysis. Screening of presumptive ESBL-GNB was done by direct inoculation of rectal swab samples on MacConkey agar (MCA; Oxoid, UK) plates supplemented with 2 µg/ml cefotaxime (MCA-C) incubated in ambient air at 37 °C for 24 h [18, 19]. CHROMagar ESBL plates (BD BBL™ CHROMagar™ ESBL, Germany) were used for primary identification while physiological and biochemical characteristics (lactose fermentation; production of CO_2_, H_2_S, indole, urease and oxidase; motility; and utilization of citrate) were used for secondary identification of isolates to species level as reported [20]. Discs combination method (ceftazidime 30 µg and cefotaxime 30 µg with and without clavulanic acid 10 µg) was used for phenotypic confirmation of ESBL production in *E. coli*, *K. pneumoniae* and *K. oxytoca* as recommended by Clinical and Laboratory Standards Institutes (CLSI) [21]. All isolates were archived in vials containing 20% glycerol in brain heart infusion (BHI; Oxoid, UK) broth and stored at − 40 °C untill molecular analysis.

#### Determination of minimum inhibitory concentration (MIC) of cefotaxime

The minimum inhibitory concentrations (MICs) of ESBL-GNB to cefotaxime were determined using agar incorporation method [22, 23] on Mueller Hinton agar (MHA; Oxoid, UK) plates supplemented with 4 µg/mL, 8 µg/mL and 16 µg/mL cefotaxime. Inoculated plates were incubated in ambient air at 37 °C for 18–24 h. The MICs were recorded as greater than the highest concentration tested or the lowest concentration when no growth occurs on any of the agar plates.

#### DNA extraction and molecular detection of bla_CTX-M-1_ group

Out of 128 isolates, 98 ESBL-GNB (73 *E. coli*, 18 *K. pneumoniae* and 7 *K. oxytoca*) were selected and successful recovered for molecular characterization of the *bla*_CTX-M-1_ group. Selection of isolates for molecular characterization was limited by the availability of PCR reagents. The isolates were sub-cultured on plain MCA (Oxoid, UK) plates followed by crude DNA extraction using boiling method as previously described [24]. Out of five major phylogenetic groups of CTX-M genes (CTX-M-1, CTX-M-2, CTX-M-8, CTX-M-9, and CTX-M-25), we chose to test only for CTX-M-1 due to predominance of its members especially the *bla*_CTX-M-15_ and from insufficient resources. PCR amplifications of the *bla*_CTX-M-1_ group was carried out in thermal cycler machine (PCR Gene AmpR System) with primers CTX-M3G-F (5′-GTTACAATGTGTGAGAAGCAG) and CTX-M3G-R (5′-CCGTTTCCGCTATTACAAAC) and procedures reported previous [25]. PCR products were electrophoresed (at 110 V for 90 min) by using 2% agarose gel which was stained by SBR-Safe DNA gel stain (ThermoFisher Scientific, UK) and visualized under UV light.

#### Quality control

Known ESBL-GNB from [26] and *E. coli* ATCC 25922 were used as control organisms.

#### Data analysis

STATA software version 13.0 was used for data analysis as per objectives of this study.

## Results

### Socio-demographic and clinical characteristics of study participants

A total of 285 patients with median age (IQR: interquartile range) of 18 (3–34) years were enrolled with the majority (53%, n = 151) being males (Table 1).

**Table 1:** Socio-demographic and clinical characteristics of study participants

Variables		Frequency (n)/median (IQR)	Percentage (%)
Median age (IQR) in years		18 (3–34)	–
Median days (IQR) of hospital stay		1 (1–3)	–
Median days (IQR) of antibiotic exposure		2 (1–3)	–
Gender			
Males		151	53
Females		134	47
Hospital of admission			
MH		29	10.2
MRH		256	89.8
Admitted ward during enrollment			
Medical		205	71.9
Surgical		80	28.1
Antibiotics use past three months			
No		148	51.9
Yes		137	48.1
Antibiotics use at enrollment			
No		107	37.5
Yes		178	62.5
On β-lactams during sampling			
No		12	6.7
Yes		166	93.3
Type of antibiotics used during sampling			
Ciprofloxacin/gentamicin		12	6.7
Penicillins		95	53.4
Cephalosporins		71	39.9
History of admission			
No		203	71.2
Yes		82	28.2
Livestock keeping			
No		253	88.8
Yes		32	11.2
HIV status			
Negative		283	99.3
Positive		2	0.7

*MH* Mazimbu Hospital, *MRH* Morogoro Regional Hospital

### Carriage of ESBL-GNB, MICs, and harboring of bla_CTX-M-1_ group in ESBL-GNB

**Fig. 1:**
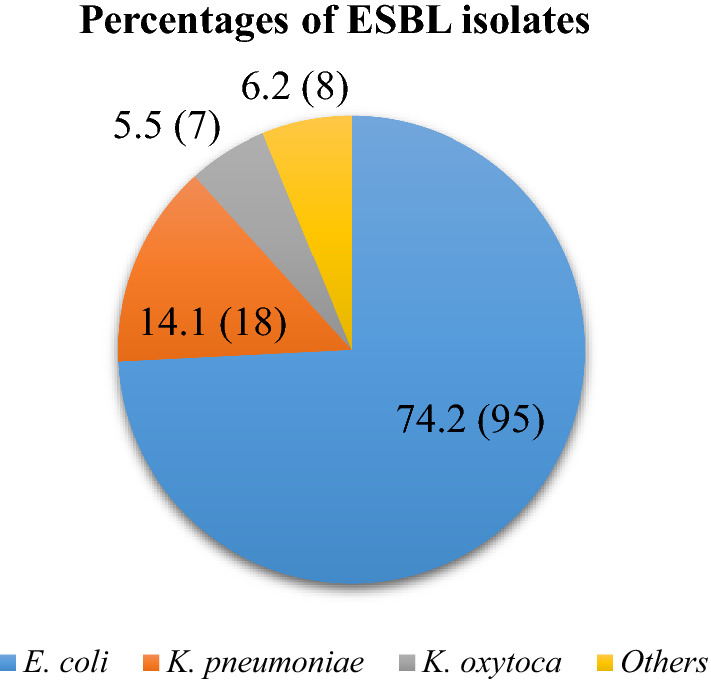
Genus and species of ESBL-GNB colonizing patients admitted at Mazimbu hospital and Morogoro regional hospital (Other isolates: *C. freundii* (n = 2), *S. marcescens* (n = 2), *Shigella* spp (n = 2), *Providencia* spp (n = 2) and *Acinetobacter* spp (n = 2))

Of the 285 patients investigated, 123 (43.2%) were colonized by ESBL-GNB whereby five patients had two ESBL-GNB isolated from single rectal swab making a total of 128 isolates (Fig. 1). Of 128 ESBL confirmed isolates, 123 (96.1%) had a MIC of ≥ 16 µg/mL while the remaining 5 isolates had a MIC of ≥ 4 µg/mL. Ninety-nine ESBL-GNB tested, 93 (94.9%) carried *bla*_CTX-M-1_ group genes while the remaining five (all were *Escherichia coli*) had no *bla*_CTX-M-1_ group genes.

### Factors associated with rectal colonization by ESBL-GNB

On multivariable logistic regression analysis controlled by age and sex: history of antibiotic use (OR: 1.83, 95% CI: 1.1–3.2, P = 0.03), being on antibiotic treatment (OR: 2.61, 95% CI: 1.5–4.53, P = 0.001), duration of hospital stay (OR: 1.2, 95% CI: 1.1–1.3, P < 0.001) and history of previous hospital admission (OR: 2.24, 95% CI: 1.2–4.1, P = 0.009) were independently found to predict ESBL-PE GNB carriage (Table 2).

**Table 2:** Factors associated with ESBL-GNB colonization

Variable		All participants (N = 285)	ESBL-GNB positive colonization N = 123 (%)	Univariable (*P* value)	Multivariable OR (95%CI)	*P* value
Median age (IQR) in years		19 (IQR: 3–33)	17 (IQR: 3–33)	0.929	0.99 [0.99–1.01]	1.000
Median (IQR) days in hospital		1 (IQR: 1–2)	2 (IQR: 1–4)	< 0.001	1.20 [1.08–1.33]	< 0.001
Antibiotic use past 3 months						
No		148	49 (33.1)	< 0.001	1 2.16 [1.22–3.84]	0.009
Yes		137	74 (54.0)			
Gender						
Females		134	51 (38.1)	0.102	1 0.81 [0.47–1.39]	0.443
Males		151	72 (47.7)			
Type of ward of admission						
Medical		205	91 (44.4)	0.502	1 1.24 [0.64–2.39]	0.526
Surgical		80	32 (40.0)			
On antibiotic use during sampling						
No		107	28 (26.2)	< 0.001	1 2.20 [1.23–3.95]	0.008
Yes		178	95 (53.4)			
Hospital admission past 3 months						
No		203	70 (34.5)	< 0.001	1 2.17 [1.17–4.01]	0.013
Yes		82	53 (64.6)			
Livestock keeping						
No		253	112 (44.2)	0.287	1 0.51 [0.22–1.19]	0.122
Yes		32	11 (34.4)			

## Discussion

This study identified a high carriage of ESBL-GNB in Morogoro regional hospital and Mazimbu hospital. The overall prevalence (43.2%) observed in this study is comparable to a study in Gabon [27]. Although the carriage in our study is relatively lower compared to (50.4%) a study conducted at Tanzanian National Hospital, Muhimbili National hospital (MNH), in Dar es Salaam [28]. Being a national referral hospital, MNH receives patients with multiple antibiotics exposure from other healthcare facilities mainly regional and zonal referral hospitals, increasing the risk of carriage of ESBL-GNB.

*E. coli* followed by *K. pneumoniae* are predominant ESBL producers colonizing patients. Similar findings were reported previous in Ethiopia, Turkey and other regions of Tanzania [15, 16, 29, 30]. Pathogenic potential of *E. coli* (e.g., *E. coli* ST131) and *K. pneumoniae* (e.g., *K. pneumoniae* ST14), and frequent acquisition of conjugative plasmids encoding for antimicrobial resistance genes (ARGs i.e., ESBL genes) facilitates rapid exchange and dissemination of ARGs in *E. coli* and *K. pneumoniae* [31]. These isolates, ESBL-GNB, colonizing patients are potentially shaded of to contaminate patient’s immediate inanimate surroundings as previous reported [32, 33]. Thus increasing the risk of exogenous source of acquiring of healthcare associated infections (HCAIs) from ESBL-GNB among vulnerable patients (immunocompromised and critically ill) associated with increased mortality from treatment failures and limited antibiotic therapeutic options [34, 35]. Therefore, this study’s findings alert for the strengthening of infections prevention and control measures and AMR surveillance in line with the Tanzania National Action Plan in order to combat AMR in the country in all tiers of health facilities [36].

This study found high proportion (94.9%) of ESBL-GNB carrying CTX-M-1 group genes colonizing patients. The CTX-M-1 group genes particularly *bla*_CTX-M-15_ are predominantly reported in clinical, colonization and environment isolates in Tanzania and elsewhere [6–8, 15, 16, 37]. Horizontal gene transfer (HGT) of mobile genetic elements (MGEs) including plasmids, transposons, and intergrons facilitates rapid dissemination and spreading of CTX-M-1 group genes, mostly in *E. coli* and *Klebsiella* spp., [8, 38–40] in the hospital environment. These findings hint the possibility of the common genetic elements or resistant strains carrying CTX-M-1 genes in healthcare facilities in Tanzania, necessitating the strengthening of IPC and antimicrobial stewardship in Tanzania.

Hospital admission, previous and current antibiotic use, and longer hospital stay significantly predicted carriage of ESBL-GNB. These findings are in consistency with other studies [5, 16, 27]. Hospital admission and longer stays increases the odds of being exposed to antibiotics mostly beta-lactams i.e., ampicillin and ceftriaxone as they make first- and second-lines of therapy [41]. Therefore, increasing antimicrobial selection pressure favoring the proliferation of resistant bacterial strains colonizing patients’ gastro-intestinal tracts as observed in this study [42, 43].

Antibiotic exposure creates essential pressure which select the small fraction of resistant bacteria of the intestinal microbiota therefore giving rise to the emergency and establishment of an entirely resistant population of bacteria [42]. With poor IPC practices especially in low- and middle-income countries, these superbugs may be cross-transmitted between patients resulting to subsequent invasive infections such as BSIs, UTIs and SSTIs. Presence of these bacteria in the gut and environment may also result in exchange of resistance genes to the highly virulent bacteria making the infection difficult to treat hence high morbidity and mortality [42].

## Limitations

From limited funds and resources: ESBL isolates were conventionally identified to possible genus and species; agar dilution method was used to determine MICs for cefotaxime only; and other ESBL alleles contributing about 5–10% of ESBL genes in our setting and genetic relatedness of ESBL isolates were not determined.

## Data Availability

The datasets generated and/or analyzed during the current study are available in the department of Microbiology and Immunology repository of the Catholic University of Health and Allied Sciences-Bugando. The data can be obtained upon request to the Director of Research and Innovation of the Catholic University of Health and Allied Sciences.

## Abbreviations

ATCC: American Type Culture Collection
BSIs: Bloodstream infections
CI: Confidence interval
DNA: Deoxyribose nucleic acid
ESBL: Extended spectrum beta lactamase
ESBL-EC: Extended spectrum beta lactamase producing *Escherichia coli*
ESBL-GNB: Extended spectrum beta lactamase producing gram negative bacteria
ESBL-KP: Extended spectrum beta lactamase producing *Klebsiella pneumonia*
GNB: Gram negative bacteria
HIV: Human immunodeficiency virus
IPC: Infection prevention and control
IQR: Interquartile range
MCA: MacConkey agar
MCA-C: MacConkey agar supplemented with cefotaxime 2 µg/mL
MIC: Minimum inhibitory concentration
MRRH: Morogoro Regional Referral Hospital
OR: Odd ratio
PCR: Polymerase chain reaction
SSTIs: Skin and soft tissue infections
UTI: Urinary tract infections
